# Cerebrospinal fluid lactate level as a diagnostic biomarker for bacterial meningitis in children

**DOI:** 10.1186/1865-1380-7-14

**Published:** 2014-02-27

**Authors:** Eduardo Mekitarian Filho, Sérgio Massaru Horita, Alfredo Elias Gilio, Lise E Nigrovic

**Affiliations:** 1 Pediatric Emergency Department, University Hospital, University of Sao Paulo, and Hospital Israelita Albert Einstein, Av. Prof. Lineu Prestes, 2565 Sao Paulo, Brazil; 2 Pediatric Emergency Department, University Hospital, University of Sao Paulo, Av. Prof. Lineu Prestes, 2565 Sao Paulo, Brazil; 3 Division of Emergency Medicine, Children’s Hospital, Boston, 300 Longwood Avenue, Boston, MA, USA

**Keywords:** Bacterial meningitis, Aseptic meningitis, Cerebrospinal fluid, Lactate

## Abstract

**Background:**

Cerebrospinal fluid (CSF) lactate is a potential biomarker for bacterial meningitis in children. To this end, we performed a single-center retrospective cohort study of children from Sao Paulo, Brazil, with CSF pleocytosis to evaluate the ability of CSF lactate to distinguish between children with bacterial and aseptic meningitis. We determined the optimum cutoff point for CSF lactate using receiver-operator curve (ROC) analysis.

**Findings:**

We identified 451 children of whom 40 (9%) had bacterial meningitis. Children with bacterial meningitis had a higher median CSF lactate level [9.6 mmol/l, interquartile range (IQR) 3.2-38.5 mmol/l bacterial meningitis vs. 2.0 mmol/l, IQR 1.2-2.8 mmol/l aseptic meningitis]. A CSF lactate cutoff point of 3.0 mmol/l had a sensitivity of 95% [95% confidence interval (CI) 83-99%), specificity of 94% (95% CI 90-96%) and negative predictive value of 99.3% (95% CI 97.7-99.9%) for bacterial meningitis.

**Conclusions:**

In combination with a validated meningitis clinical prediction rule, the CSF lactate level can be used to distinguish between bacterial and aseptic meningitis in children with CSF pleocytosis.

## Findings

### Introduction

While bacterial meningitis causes significant morbidity and mortality despite advances in antibiotic therapy, aseptic meningitis is typically a benign condition requiring only supportive care [1]. Rapid differentiation between bacterial and aseptic meningitis allows early initiation of appropriate therapy for children at risk for having bacterial meningitis without overtreating low-risk children. Although available conjugate vaccinations against *Streptococcus pneumoniae* and *Neisseria meningitidis* have decreased the bacterial meningitis incidence, incomplete vaccine uptake as well as infections caused by bacterial serogroups not included in the vaccine makes bacterial meningitis a clinical concern in children, especially in resource-poor settings [2].

The gold standard for the diagnosis for bacterial meningitis is culture, which requires several days to return results [3,4]. Rapidly available biomarkers such as cerebrospinal (CSF) lactate have been studied to distinguish bacterial from aseptic meningitis before the results of bacterial cultures become available. CSF lactate levels are high in children with bacterial meningitis as lactate is produced by both bacterial anaerobic metabolism as well as ischemic brain tissue [5]. The predictive ability of CSF lactate for bacterial meningitis has been examined in two recently published meta-analyses [6,7]. In our large retrospective cohort of children with meningitis, we sought to establish the optimal CSF lactate cutoff point to accurately distinguish between bacterial and aseptic meningitis [8].

### Materials and methods

To this end, we performed a retrospective cohort study of children with meningitis between 1 month and 15 years of age who presented to a single emergency department (ED) of the University Hospital over the 12-year period (2001–2012). The study was approved by the institutional review board. Details of the study protocol have been described previously [8]. We included children who had clinical findings consistent with meningitis (e.g., fever, headache, vomiting or nausea, and neck stiffness) along with CSF white blood cells (WBC) ≥10 cells/μl [corrected for the presence of CSF red blood cells (RBC) using a standard 1:500 ratio of leukocytes to erythrocytes] and who had both a CSF lactate and CSF culture obtained. We further limited our study population to those children who had CSF lactate obtained by the treating clinician. We excluded children with any of the following: critical illnesses, any purpura, presence of a ventricular shunt or recent neurosurgery, immunosuppression and other bacterial infections requiring parenteral antibiotics. We also excluded children who had received any antibiotic pretreatment within 72 h of diagnostic lumbar puncture as pretreatment can render cultures falsely negative.

We reviewed the medical records for all eligible children and abstracted relevant clinical and laboratory factors for all study patients. The CSF lactate level was measured by the hospital clinical laboratory using standard enzymatic methods (ADIVA Chemistry, Siemens, Bayswater Victoria, Australia).

We defined a case of bacterial meningitis as a child with either a positive CSF culture or CSF pleocytosis with a positive blood culture for a bacterial pathogen [9]. We defined a case of aseptic meningitis as a child with meningitis who had not received antibiotics prior to diagnostic lumbar puncture who had a negative CSF bacterial culture [9].

For statistical analysis, we first selected a range of cutoff points for the CSF lactate biomarker to distinguish bacterial from aseptic meningitis. We reported the sensitivity, specificity and negative predictive value (NPV) for bacterial meningitis of each CSF lactate cutoff point. Next, we generated a receiver-operator curve (ROC) to visually represent the trade-off between sensitivity and specificity. We utilized ROC curve analysis to select the optimal CSF lactate cutoff point to minimize both the number of false positives (children with aseptic meningitis with CSF lactate above the chosen cutoff point) as well as false negatives (children with bacterial meningitis with CSF lactate below the cutoff point). A Mann–Whitney U-test was performed to compare lactate levels between children with bacterial and aseptic meningitis. We used PASW Statistics (version 21.0, Chicago, IL, 2012) for all statistical analyses.

### Results

We identified 494 children with meningitis who met the study criteria, of which 43 (9%) did not have a CSF lactate analysis performed. Of the 451 study patients, the mean age was 4.9 years [median 4.0 years;interquartile range (IQR) 0.8-7.3 years], and 306 (68%) were male. Of the 40 children with bacterial meningitis (9% of study patients), the two most common bacterial pathogens were *Neisseria meningitidis* (55% of bacterial meningitis cases) and *Streptococcus pneumoniae* (33% of cases). Of the 22 children with *Neisseria meningitidis* meningitis*,* 8 had a positive CSF culture alone, 9 had positive CSF and blood cultures, and 5 had a positive blood culture with CSF pleocytosis. Of the 13 children with *Streptococcus pneumoniae* meningitis, 2 had a positive CSF culture alone, 3 had positive CSF and blood cultures, and 8 had a positive blood culture with CSF pleocytosis. The remaining five children with bacterial meningitis had a positive CSF culture.

Children with bacterial meningitis had CSF lactate concentrations (Table 1). Each step-wise increase in the CSF lactate cutoff point lowered the sensitivity, but increased the specificity for bacterial meningitis (Table 2).

**Table 1:** CSF lactate concentration in children with bacterial and aseptic meningitis

	**Bacterial meningitis**	**Aseptic meningitis**
	**(*n*** **= 40)**	**(*n*** **= 411)**
Mean CSF lactate, mmol/l	22.1	2.8
(SD)	(±14.1)	( ±2.2)
Median CSF lactate, mmol/l	9.6	2.0
(interquartile range)	(3.2–38.5)	(1.2–2.8)

**Table 2:** The diagnostic accuracy of CSF lactate for bacterial meningitis by cutoff point

**CSF lactate (mmol/l)**	***n*** **= 451**	**Sensitivity**	**Specificity**	**Negative predictive value (NPV)**
	***n *** **(%)**	***n (*%) (95% CI)**	***n *** **(%) (95% CI)**	***n*% (95% CI)**
≥1.0 mmol/l	442 (98)	100 (91.2-100)	2.8 (1.3-5.2)	100 (66.4-100)
≥1.5 mmol/l	388 (86)	100 (91.2-100)	25.2 (20.5-30.2)	100 (95.6-100)
≥2.0 mmol/l	246 (55)	97.5 (86.8-99.9)	55.2 (49.6-60.7)	99.4 (97.0-100)
≥2.5 mmol/l	102 (23)	95.0 (83.1-99.4)	85.6 (81.3-89.2)	99.3 (97.5-99.9)
≥3.0 mmol/l	56 (12)	95.0 (83.1-99.4)	93.6 (90.3-96.0)	99.3 (97.7-99.9)
≥3.5 mmol/l	47 (10)	90.0 (76.3-97.2)	96 (93.3-97.9)	98.7 (96.8-99.7)
≥4.0 mmol/l	34 (7.5)	82.5 (67.2-92.7)	96.3 (93.7-98.1)	97.8 (95.6-99.1)
≥4.5 mmol/l	30 (6.6)	72.5 (56.1-85.4)	96.6 (94.0-98.3)	96.6 (94.0-98.3)
≥5.0 mmol/l	26 (5.8)	72.5 (56.1-85.4)	97.5 (95.2-98.9)	96.2 (94.1-98.3)

**Figure 1:**
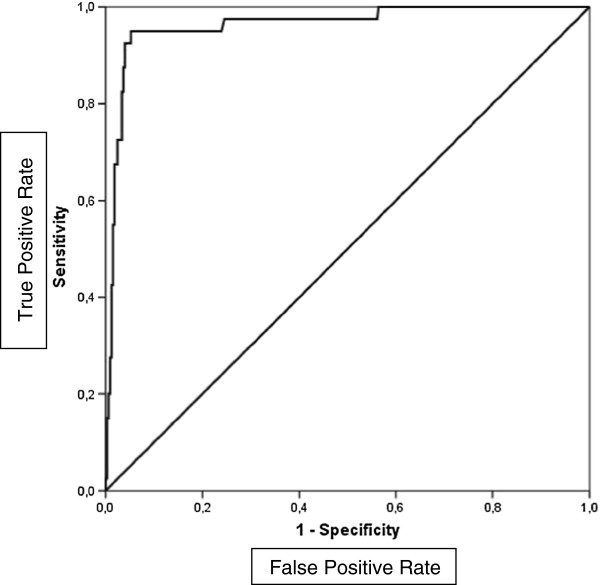
Receiver-operating curve for CSF lactate to distinguish bacterial from aseptic meningitis.

Next we present a ROC curve for CSF lactate for bacterial meningitis (Figure 1). Using ROC analysis, we selected an optimal cutoff point for CSF lactate of 3.0 mmol/l to distinguish between bacterial and aseptic meningitis [area under the curve 0.96; 95% confidence interval (CI) 0.93-0.99]. CSF lactate ≥3.0 mmol/l had a sensitivity of 95.0% (95% CI 83.1–99.4%), a specificity of 93.6% (95% CI 90.3-96.0%) and NPV of 99.3% (95% CI 97.7-99.9%) for bacterial meningitis.

Two children with bacterial meningitis had a CSF lactate value < 3.0 mmol, but both had a positive CSF gram stain as well as peripheral and CSF leukocytosis. One of these children had *Staphylococcus aureus* meningitis and the other *Streptococcus pyogenes* meningitis. Both children were hospitalized and received broad-spectrum antibiotics, with good clinical outcomes.

### Discussion

In our large retrospective cohort of children with meningitis, CSF lactate ≥3.0 mmol/l accurately distinguished between bacterial and aseptic meningitis. However, two children (5% of children with bacterial meningitis) had CSF lactate below the cutoff point. Therefore, CSF lactate should not be used alone but rather combined with a validated clinical prediction model such as the Bacterial Meningitis Score [8-10]. Importantly, this prediction model appropriately identified both children with bacterial meningitis and CSF lactate <3.0 mmol/l.

Two recent meta-analyses evaluated the ability of CSF lactate to distinguish aseptic from bacterial meningitis [6,7]. These two studies included 38 unique studies with between 15 and 201 patients per study. Using a CSF lactate cutoff point between 2.1 and 4.4 mmol/l, the aggregated sensitivity of CSF lactate for bacterial meningitis was 93% or 96% and specificity 94% or 96%, respectively. Our findings were similar, but our study was larger than any of the included studies. Additionally, our study was the only pediatric study conducted in an ED setting where initial management decisions for children with meningitis must occur.

The management of children with meningitis may differ in resource-poor settings asthe incidence of bacterial meningitis may be considerably higher and clinicians may have limited access to diagnostic laboratories [11]. Available hand-held testing platforms can provide point-of-care testing for CSF lactate. In a small study conducted in Uganda, CSF lactate analysis using a hand-held device had good sensitivity and specificity for bacterial meningitis [12]. Larger, likely multi-centered, studies are needed to further evaluate the diagnostic accuracy of CSF lactate in resource-poor settings.

Our study has several limitations. First our study was retrospective, and we could not assess general appearance of the children. Children who appear unwell at presentation require inpatient therapy regardless of the risk of bacterial meningitis. Second, not all children with aseptic meningitis had a specific infectious etiology identified. However, we reliably excluded bacterial meningitis in these children as all had a CSF bacterial culture with no antibiotic pretreatment within 72 h of diagnostic LP. Third, not all children had CSF lactate obtained and were not included in our study. In fact, the proportion of children who had CSF lactate increased over the study time period. However, the clinical and laboratory presentations of children who did and did not have CSF lactate obtained were similar (data not shown). Last, we had a relatively small number of children with bacterial meningitis, which limited our ability to measure the diagnostic accuracy of CSF lactate. We were also unable to compare CSF lactate by bacterial meningitis pathogen.

The findings of this study suggest that CSF lactate is a reliable biomarker to distinguish between bacterial meningitis from aseptic meningitis, albeit with imperfect sensitivity. In combination with other validated clinical prediction models, CSF lactate can assist with clinical decision-making for children with meningitis.

### Informed consent

Written informed consent was obtained from the patient’s guardian/parent/next of kin for the publication of this report and any accompanying images.

## Competing interests

The authors declare that they have no competing interests.

## Authors’ contribution

Dr. MF was responsible for study conception, carried out data collection and analysis, as well as manuscript writing and editing. Dr. SH carried out data collection and manuscript editing. Dr. AG was responsible for manuscript writing, study conception and manuscript analysis. Dr. LN was responsible for data analysis, paper writing and manuscript editing. All authors read and approved the final manuscript.
